# Mr. Marson, on Vaccination

**Published:** 1806-07

**Authors:** W. Marson

**Affiliations:** Worksop Nots


### *To the Editors of the Medical and Physical Journal.*

Gentlemen,

THE letter I troubled you with in February last you have been pleased to pronounce critical and controversial, and therefore not suitable for your valuable publication; the substance of that letter (the improvement of vaccination) I am now the more anxious to be inserted, as I have since perceived in the Medical Journal, some of the hints which you will allow I offered you. I do not suppose they were surreptitiously published, but that they are of the more importance as having been contemplated by others.—You will have the goodness then to favour the following observations vations with insertion, as you will find the former exceptionable parts are done away.

I think sufficient attention has not been paid by the Jennerian Society to the arguments of those who have written in favour of variolous inoculation, and I would recommend the committee to examine well their mode of practice, and if a regimen is pursued or medicines prescribed, how far any appearances of the inoculated arm appear to be altered by it.

Several respectable practitioners, in this way, have declared their particular success; if then, by an examination of their reasons for the practice of regimen and medicine, the committee do not find themselves dissatisfied, it would be fair, in my opinion, to recommend the application of a like plan in vaccine inoculation. I have ever considered a disease which is to take the place of the small-pox, and observe so nearly its laws in its progress, should require the same precautions for insuring success; and I believe few variolous inoculators have rested on the mere insertion of the matter.—I submit then to the committee to consider how far medicine and regimen would be beneficial in vaccine inoculation, and if any unfavourable interruption to the progress of the disease, or any unpleasant consequence, can thus be prevented. The inoculator should examine the constitution, and see if there be any herpetic eruption or scrophulous appearance, if the intestinal canal is in a right state, &c. In fine, let the constitution be made healthy if it is not so, and not add a disease to an already diseased habit; and in this way, and in this only, do I conceive justice can be done to Dr. Jenner and his valuable discovery. I do assure you, gentlemen, on this ground I was very early sceptical as to the uninterrupted success of vaccine inoculation; and I am persuaded the disputatious will be soon silent, if what I hint has the honour to be attended to.

It is said by some, but which Dr. Jenner disallows, that vaccine inoculation is not interrupted by herpetic eruptions, and that they die away with it; still I think we should be justifiable in giving medicine for these eruptions, as I cannot see what advantage can be gained as to the inoculation, "by all the herpetic eruptions inflaming with the inoculated arm."

There were some practitioners of variolous inoculation who never used medicine or regimen, and I believe they were led into this error, by thinking that the celebrity of Sutton depended on cold air; but it should be remembered

Baron Dimsdale was also famous in his practice, and they were considered as inoculating differently. These rival practitioners were both eminent, and yet we cannot infer they differed as to the mode of taking the air or its temperature; no, they gave medicine and observed a regimen, particularly Sutton, and he watched the progress of the inoculated arm, and if this did not make the way he expected or wished, appropriate medicines were given; it was only during the eruptive fever Sutton laid such stress on cold air.

I lament to see the delay and disappointment in vaccination occasioned by the frequent failures of the inoculation taking place; some of your correspondents state the very many times they have inoculated before it has taken effect; and Dr. Walker, in your last number, speaks of inoculating twenty-nine times before success attended his friend's indefatigability. Indeed, gentlemen, this appears to me a very serious matter, as at least it must be two months before he had done with the patient, who all this time was liable to take the small-pox. By giving me a little credit, I think you will find this unpleasant circumstance of frequent vaccination in the same patient seldom recorded; the failures in general I conceive to be owing to the non-absorption of the matter, and that by putting the absorbents in a hungry state, a failure will seldom happen.

I am, &c.

W. MARSON.

*Worksop Nots, June* 10, 1806.

